# Joint Cardioprotective Effect of Vitamin C and Other Antioxidants against Reperfusion Injury in Patients with Acute Myocardial Infarction Undergoing Percutaneous Coronary Intervention

**DOI:** 10.3390/molecules26185702

**Published:** 2021-09-21

**Authors:** Ramón Rodrigo, Juan Carlos Prieto, Rubén Aguayo, Cristóbal Ramos, Ángel Puentes, Abraham Gajardo, Emiliano Panieri, Catalina Rojas-Solé, José Lillo-Moya, Luciano Saso

**Affiliations:** 1 Molecular and Clinical Pharmacology Program, Faculty of Medicine, Campus Norte, Institute of Biomedical Sciences, University of Chile, Avda. Independencia 1027, Santiago 8380000, Chile; jprieto@u.uchile.cl (J.C.P.); catalinarojass@ug.uchile.cl (C.R.-S.); joselillo@ug.uchile.cl (J.L.-M.); 2 University of Chile Clinical Hospital, Campus Norte, Carlos Lorca Tobar 999, Independencia, Santiago 8380456, Chile; cramos@u.uchile.cl (C.R.); abrahamgajardo@hotmail.com (A.G.); 3 Cardiology Unit, Department of Medicine, Occident Division, San Juan de Dios Hospital, Avenida Portales 3239, Santiago 8500000, Chile; raguayo@minsal.cl (R.A.); apuentescdt@yahoo.com (Á.P.); 4 Department of Physiology and Pharmacology “Vittorio Erspamer”, Faculty of Pharmacy and Medicine, Sapienza University, P.le Aldo Moro 5, 00185 Rome, Italy; emiliano.panieri@isprambiente.it (E.P.); luciano.saso@uniroma1.it (L.S.)

**Keywords:** vitamin C, antioxidants, cardioprotection, ischemia-reperfusion, percutaneous coronary intervention

## Abstract

Percutaneous coronary intervention (PCI) has long remained the gold standard therapy to restore coronary blood flow after acute myocardial infarction (AMI). However, this procedure leads to the development of increased production of reactive oxygen species (ROS) that can exacerbate the damage caused by AMI, particularly during the reperfusion phase. Numerous attempts based on antioxidant treatments, aimed to reduce the oxidative injury of cardiac tissue, have failed in achieving an effective therapy for these patients. Among these studies, results derived from the use of vitamin C (Vit C) have been inconclusive so far, likely due to suboptimal study designs, misinterpretations, and the erroneous conclusions of clinical trials. Nevertheless, recent clinical trials have shown that the intravenous infusion of Vit C prior to PCI-reduced cardiac injury biomarkers, as well as inflammatory biomarkers and ROS production. In addition, improvements of functional parameters, such as left ventricular ejection fraction (LVEF) and telediastolic left ventricular volume, showed a trend but had an inconclusive association with Vit C. Therefore, it seems reasonable that these beneficial effects could be further enhanced by the association with other antioxidant agents. Indeed, the complexity and the multifactorial nature of the mechanism of injury occurring in AMI demands multitarget agents to reach an enhancement of the expected cardioprotection, a paradigm needing to be demonstrated. The present review provides data supporting the view that an intravenous infusion containing combined safe antioxidants could be a suitable strategy to reduce cardiac injury, thus improving the clinical outcome, life quality, and life expectancy of patients subjected to PCI following AMI.

## 1. Introduction

During the last two decades, heart disease has remained the leading cause of death worldwide, accounting for 16% of deaths from all causes [1]. In this line, heart attacks and strokes account for up to four out of five deaths [2]. Acute myocardial infarction (AMI) occurs through the complete or partial lumen occlusion of a branch of coronary circulation. Atheroma plaques are vulnerable to rupture or erosion, thereby causing thrombotic alterations that result in blocking blood flow to the myocardial tissue [3]. Percutaneous coronary intervention (PCI) is an invasive non-surgical therapeutic procedure whose aim is to improve the blood supply directed to the ischemic tissue. For this purpose, the most common method is the inflation of the narrow segment or the deployment of a stent to keep the artery open and regain its volume [4]. At present, PCI remains the gold standard therapy for treating blood occlusion, particularly in AMI. Several trials have shown that PCI is more effective than thrombolytic therapy to restore blood flow in patients with ST-segment elevation myocardial infarction [5]. However, paradoxically, the restoration of blood flow in the ischemic myocardium also induces additional injury. This phenomenon was therefore termed myocardial ischemia reperfusion injury (MIRI). Subsequent studies in animal models of AMI have suggested that myocardial reperfusion accounts for up to 50% of the final size of a myocardial infarct [6]. The key role of oxidative stress in the pathophysiology of MIRI is due to the burst of reactive oxygen species (ROS) occurring immediately after the onset of reperfusion. In order to abrogate this injurious mechanism, numerous antioxidant treatments have aimed to reduce MIRI by enhancing the antioxidant defense system through the administration of vitamin C (Vit C), vitamin E (Vit E), N-acetylcysteine (NAC), deferoxamine (DFO), and polyphenols, among others. However, these therapies have led to suboptimal results in animal models and have not produced consistent effects when tested in clinical trials. Consequently, to date, there has been no available therapy against MIRI for these patients, and it remains a challenge to develop an effective therapy.

Vit C is involved in numerous physiological processes, and is known to exert pleiotropic therapeutic effects in a variety of human pathologies, including cardiovascular diseases [7]. Accordingly, it has been used to reduce oxidative stress in ischemia/reperfusion (I/R) processes, both in animal models and clinical trials, but its benefit in reducing MIRI remains uncertain. The lack of fundamental knowledge about the pharmacological properties of Vit C has led to suboptimal design, misinterpretations, and the erroneous conclusions of clinical trials [8]. All of this has disallowed the drawing of definitive conclusions supporting a therapeutic use for Vit C. Nevertheless, there are some studies showing beneficial effects. Thus, Khan et al. [9] carried out a systematic review of randomized controlled trials including 1185 patients selected from 371 publications reporting cardioprotective effects of Vit C during angioplasty, but also showing mixed results among the different studies. The administration of Vit C prior to PCI reduced cardiac injury biomarkers, as well as inflammatory markers and ROS formation. However, despite improvements of functional parameters, such as the trend shown by left ventricular (LV) ejection fraction (LVEF) and the telediastolic left ventricular volume, the association with Vit C was inconclusive. This could be due to an approach that points primarily towards the ROS scavenger ability of Vit C, rather than its mechanistic effects, such as its pharmacokinetics, pharmacodynamics, interaction with metal ions and their recycling. Moreover, AMI has a multifactorial nature, causing the death of cardiomyocytes and other heart tissue cell types through different pathways, such as apoptosis, necrosis, autophagy, and ferroptosis [10]. Accordingly, it has been previously proposed that cardioprotection for MIRI requires a synergistic or additive multitarget therapy [11]. This study provides data supporting the suitable use of an intravenous infusion containing combined safe antioxidants to reduce reperfusion cardiac injury. This enhanced cardioprotective effect might improve the clinical outcome, life quality, and life expectancy of AMI patients undergoing PCI.

## 2. Ischemia-Reperfusion Induced Cardiac Injury and Oxidative Stress

### 2.1. Oxidative Stress Development

Oxidative stress occurs due to an imbalance between the production of oxidant species and the activity of the antioxidant system, in favor of the first, which has been involved in the pathophysiological mechanisms of various cardiovascular pathologies [12]. Indeed, various reactive species are generated within the cells, either by enzymatic or non-enzymatic pathways, fulfilling an important physiological role in the regulation of important cellular functions through redox signaling. Thus, the physiological role of these species depends on their concentration at the steady state, the specific site, and the extent of their generation over time. According to their chemical nature, we can distinguish reactive oxygen species derived from oxygen, nitrogen, or sulfur, with the first two types being particularly relevant for human pathology. Reactive oxygen species include superoxide anion radical (O_2_**^.−^**), hydrogen peroxide (H_2_O_2_), hydroxyl radical (**^.^**OH), and oxygen singlet (^1^O_2_). In turn, reactive nitrogen species are nitric oxide radical (NO**^.^**), peroxynitrite anion (ONOO^−^), and nitrogen dioxide radical (NOO**^.^**). The main sources of ROS are physically located within the mitochondria, wherein physiological or even pathological amounts are generated by the electron transport chain complexes due the incomplete reduction of molecular oxygen, or by the metabolic enzymes of the Krebs cycle, with the 2-oxoglutarate dehydrogenase complex being the one with the highest production rates [13]. Other relevant enzymatic sources with cell-type and compartment-specific localization include reduced nicotinamide adenine dinucleotide phosphate (NADPH) oxidase (NOX), xanthine oxidase (XO), uncoupled endothelial nitric oxide synthase (un-eNOS), lipoxygenase/cyclo-oxygenases, and myeloperoxidase (MPO), among others [14]. In addition, ROS can be formed through non-enzymatic pathways, such as chemical redox reactions involving ferrous iron, hydrogen peroxide, and the superoxide anion (Fenton and Haber–Weiss reactions) leading to the production of OH, the most reactive and harmful one. Increased ROS concentrations at the steady state can potentially cause damage to biomolecules, such as lipids, proteins, and DNA, thus altering their structure and normal functioning. The pathophysiological effects of this damage range from an inflammatory state to several forms of cell death [15].

### 2.2. Antioxidant Defense System

In order to comply with the oxidative challenges, the cell has evolved a wide array of mechanisms that collectively constitute the antioxidant system. Its main function is to constantly shape the steady-state levels of ROS production to either support redox signaling events or to counteract the uncontrolled elevation of ROS levels, thus preventing or repairing the oxidative damage. This defense system works through both enzymes and other non-enzymatic molecules, being superoxide dismutases (SODs), catalase (CAT), peroxiredoxins (PRXs), thioredoxins (TRXs), glutaredoxins (GRXs), and glutathione peroxidases (GPXs), the main enzymes and the first line of defense against oxidative damage [14]. On the other hand, non-enzymatic antioxidant molecules are represented by endogenous components, such as reduced glutathione (GSH), NADPH, and exogenous molecules including Vit C, Vit E, carotenoids, flavonoids, and polyphenols, among others [14,16]. It is of interest to mention that the antioxidant enzymatic response is largely mediated by the nuclear factor-erythroid 2-related factor 2 (Nrf2) pathway, wherein the exposure to various oxidants induces the dissociation of the inhibitory Kelch-like ECH-associated protein 1 (Keap1) subunit from the Nrf2 protein, allowing the latter to be translocated into the nucleus. Here, Nrf2 binds to the site of antioxidant response elements (AREs), stimulating the transcription of genes that code for antioxidant enzymes, such as CAT, GPX, heme oxygenase-1 (HO-1), glutathione synthase (Gclc and Gclm), and glutathione reductase (GR), thereby contributing to the maintenance of the cellular redox balance [17,18]. The relevance of this pathway in cardioprotection has been suggested by studies reporting that Nrf2 knockout mice have a larger infarct size in response to regional ischemia/reperfusion events, which was decreased by ischemic preconditioning strategies [19]. In addition, Zhang et al. [20] reported that the use of preconditioning strategies induces cardioprotection through the Nrf2 pathway, increasing the enzymatic antioxidant response and the GSH/GSSG index in rabbit hearts subjected to MIRI.

As mentioned previously, ROS can have physiological or harmful functions depending on their concentration and temporal generation. In low or moderate concentrations they participate in the response processes to damage, such as defending against infectious agents and regulating the function of a number of cellular signaling systems [21], though they would even fulfill an indirect antioxidant function at H_2_O_2_ concentrations between 1 and 10 nmol/L via the Nrf2 pathway [22]. On the other hand, at concentrations greater than 100 nmol/L, the nuclear factor kappa-light-chain-enhancer of the activated B cells (NF-κB) pathway is activated, which is the major mediator of cytokine effects in the heart, and is involved in damaging processes [14,22].

### 2.3. Pathophysiology of Cardiac Ischemia/Reperfusion Injury

Patients with AMI present a complete arrest of blood flow in specific regions of the heart, with PCI therapy being the gold standard procedure for the blood flow restoration. However, both the ischemia and reperfusion processes cause multiple energetic and biochemical changes in the cells of the myocardial tissue. These effects have been associated with multiple complications, such as lethal reperfusion, no-reflow phenomenon, myocardial stunning, and reperfusion arrhythmias [23].

The absence of oxygen caused by the ischemic period induces a decrease in adenosine triphosphate (ATP) synthesis due to a lower activity of oxidative phosphorylation, which is accompanied by an increased anaerobic metabolism, thus increasing the formation of lactic acid [24,25] and decreasing the metabolic activity of the Krebs cycle. There is also a decrease in intracellular pH that, concomitant with the ATP depletion, causes a decrease in cardiac contractile activity [26] and multiples ionic changes that culminate in a cytosolic overload [27]. This is due to the overwhelming of the sarcoplasmic reticulum Ca^2+^-ATPase that, under these circumstances, is unable to counteract the increased cytosolic Ca^2+^ concentration [28]. In addition, xanthine dehydrogenase is converted to XO, a source of O_2_**^.^**^−^ that induces oxidative stress, increasing intracellular calcium, and the mitochondrial permeability transition pore (mPTP) is inhibited as a result of acidic conditions [29].

**Figure 1:**
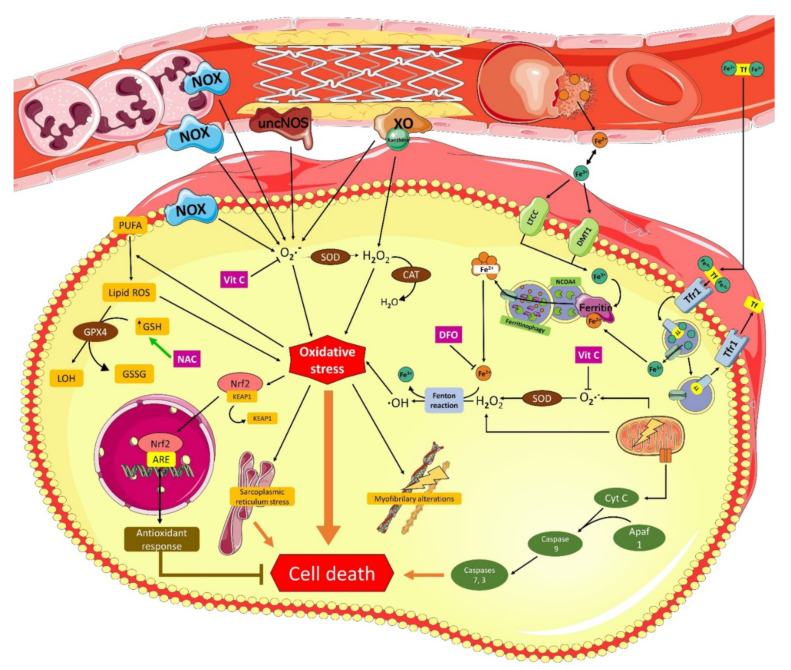
Molecular mechanisms of the deleterious effects of myocardial reperfusion injury and proposed target of combined antioxidant therapy: Apaf1, apoptosis protease-activating factor-1; ARE, antioxidant response element; CAT, catalase; Cyt C, cytochrome c; DFO, deferoxamine; DMT1, divalent metal transporter 1; GPX, glutathione peroxidase; GSH, reduced glutathione; GSSG, oxidized glutathione; KEAP1, Kelch-like ECH-associated protein 1; LTCC, L-type calcium channel; LOH, lipid alcohols; NAC, N-acetyl-L-cysteine; NCOA4, nuclear receptor coactivator 4; Nrf2, nuclear factor-erythroid 2-related factor 2; NOX, reduced nicotinamide adenine dinucleotide phosphate oxidase; PUFA, polyunsaturated fatty acids; ROS, reactive oxygen species; SOD, superoxide dismutase; Tf, transferrin; TfR1, transferrin receptor protein 1; Vit C, vitamin C; XO, xanthine oxidase.

During reperfusion, blood flow restoration leads to the recovery of mitochondrial respiration and intracellular pH [24]. As a consequence, an ROS burst occurs during the first minutes following the onset of reperfusion [30]. This event has been considered one the key contributing factors accounting for cardiac tissue injury by I/R, as it is able to trigger mechanisms of cell death in different cell types of cardiac tissue [15,31]. Indeed, the main sources leading to an increased ROS production during reperfusion correspond to the impaired mitochondrial electron transport chain, namely NOX in neutrophils and XO and un-eNOS in endothelial cells [32] (Figure 1). However, other potential sources have also been described, such as cytochrome P450, lipoxygenase, cyclooxygenase, and monoamine oxidase [33]. Furthermore, during the MIRI process, iron mobilization occurs, which increases the concentrations of the labile iron pool that, in turn, can promote the occurrence of Fenton reactions and the production of ·OH (Figure 1). Several pathways can contribute to an increase in the cytosolic free iron that has been described in MIRI, such as ferritinophagy [34], polyol pathway [35], myocardial hemorrhage [36], Fe-S cluster [37], and heavy ferritin chain [38], all of which would finally induce ferroptosis in cardiac tissue [39].

In addition, it was shown that the opening of mPTP occurs within the first few minutes following the onset of reperfusion [40], possibly due to both the burst of oxidative stress and the restoration of pH as the main contributing factors [41,42], which initially remains inhibited by acidic conditions during ischemia [29]. In this same line, a previous study showed that the early induction of temporary acidosis during reperfusion reduced infarct size in dogs [43]. However, the role of the molecular identity of mPTP is still a matter of discussion, where proteins such as adenine nucleotide translocase and FOF1-ATP synthase have been proposed to be involved, but the capacity for inducing cell death remains unclear [44,45]. Nevertheless, mPTP plays a crucial role in the pathogenesis of MIRI and has been described as a potential pharmacological target, since its inhibition was shown to reduce the infarct size by 40–50% [46,47,48,49].

Myocardial ischemia-reperfusion injury has also been significantly linked to inflammation, although it is not yet clear whether the participation of the inflammatory response that accompanies AMI contributes to the pathogenesis of reperfusion injury or whether it is just an epiphenomenon [50,51]. However, the upregulation of adhesion molecules (P-selectin, CD11/CD18, and ICAM-1) in cardiomyocytes and cytokines (TNFα, IL-1, IL-6, IL-8, NAP-1, PAF, and MIP-2), and the complement during reperfusion, has been reported, promoting the arrival of neutrophils to the infarcted areas of the myocardium [52]. Neutrophils release more than 20 different proteolytic enzymes and are a major ROS source by generating superoxide anions through NOX, thus they would have an important role in the MIRI [52]. Moreover, the increase in the formation of inflammatory molecules has been linked to the activation of the NF-κB pathway due to cytokines and oxidative stress produced by the ROS burst during reperfusion [51,53,54,55]. Furthermore, it has recently been shown that cell death by ferroptosis, as a result of I/R in a mouse heart transplantation model, initiates the recruitment of neutrophils through the TLR4/TRIF signaling pathway [56].

## 3. Cardioprotection by Vitamin C

**Figure 2:**
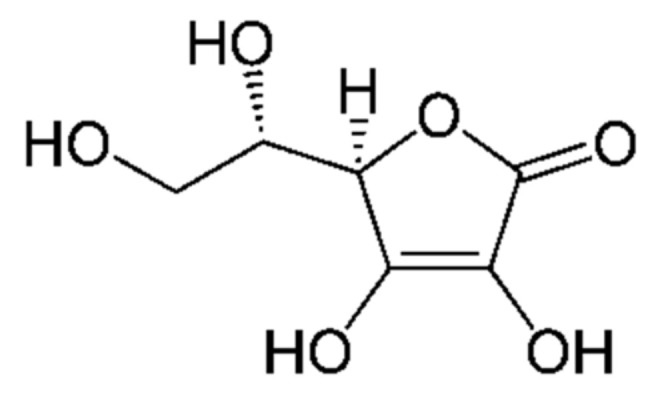
Molecular structure of vitamin C.

**Figure 3:**
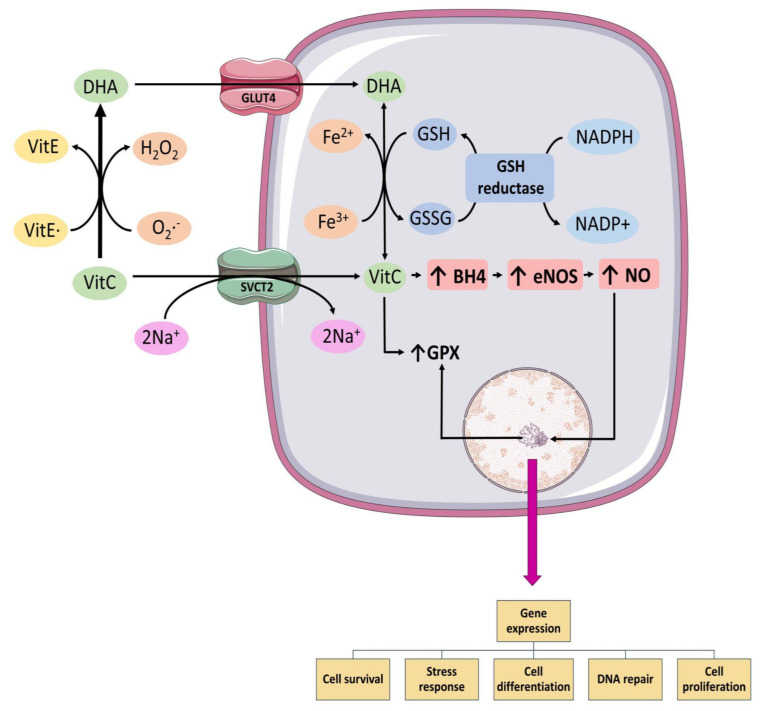
Mechanism of Vit C uptake and its effects in the cell: Reduced form of Vit C is transported through sodium-dependent transporter (SVCT2) and oxidized form, dehydroascorbic acid (DHA), is transported through GLUT4. Inside the cell, DHA is reduced back to Vit C. Vit C increases levels of tetrahydrobiopterin (BH4), levels which are a cofactor for endothelial nitric oxide synthase (eNOS) coupling and important for nitric oxide (NO) synthesis. AA, ascorbic acid; BH4, tetrahydrobiopterin; DHA, dehydroascorbic acid; eNOS, endothelial nitric oxide synthase; GLUT 4, type 4 glucose transporter; GPX, glutathione peroxidase; NO, nitric oxide; SVCT2, sodium-dependent vitamin C transporter 2; Vit E, α-tocopherol: Vit E**^.^**, α-tocopheroxyl radical.

Vitamin C (Figure 2, Table 1) is a water-soluble antioxidant compound, which fulfills essential functions in humans by being a component of the antioxidant defense system. It reduces compounds such as free radicals through its electron donating capacity, being oxidized to dehydroascorbate. Normal plasma Vit C levels are around 50–70 μmol/L [8] and are not synthesized by the human body, therefore its intake through food, such as fruits and vegetables, is the prevalent source for humans. In addition, it can be administered as a supplement, orally or intravenously. As Vit C is a water-soluble molecule, it needs transporters to be able to exert its effects within the cell. Two Na^+^-dependent transporters (SVCT1 and SVCT2) have been described as those responsible for the intracellular uptake of Vit C, while dehydroascorbate enters through GLUT family transporters, such as GLUT1, GLUT3, and GLUT4 [57] (Figure 3). Therefore, it is important to mention that human myocardium contains GLUT1, GLUT3, and GLUT4 [58].

From early studies, the use of Vit C has been proposed to counteract the oxidative stress produced by the cardiac I/R process in order to reduce the heart damage (Table 1), where it has been described that patients undergoing PCI suffer from a depletion of Vit C [59]. Several in-vitro experiments have shown the benefits of the ROS-scavenging capacity of Vit C [60], while its intra-arterial administration in high doses was found to suppress the in-vivo effects of superoxide anions on vascular endothelial dysfunction in subjects with essential hypertension [61,62]. A study showed that AMI patients undergoing thrombolysis had decreased SOD enzyme activity along with increased XO enzyme activity and malondialdehyde (MDA) levels after reperfusion, which was restored to normal or near-normal levels by oral administration of Vit C after reperfusion [63]. Clinical trials have also been carried out wherein post-perfusion Vit C has been used in patients undergoing PCI. However, although they have mainly achieved the objective of reducing the biomarkers of oxidative stress, they have not been able to reduce the infarct size [9].

### 3.1. Parameters Studied in Clinical Trials

The results obtained from the administration of Vit C to reduce post-PCI injury can be described based on four parameters: First in the biomarkers of oxidative stress and inflammation, benefits in cardiac function, reduction of cardiac damage, and finally the infarct size. These will be discussed below.

#### 3.1.1. Oxidative Stress and Inflammation Biomarkers

The measurement of oxidative stress biomarkers is one of the main parameters that are analyzed in clinical trials and studies in animals and cell cultures, assessing the effects of Vit C administration (Table 1). To this extent, different biomarkers can be used, among which those organic molecules are formed upon ROS attack to biomolecules such as lipids, proteins, or DNA and those that are related to the antioxidant capacity of the patient at the plasma and cellular levels. Clinical trials that have administered Vit C to reduce reperfusion injury after PCI have shown favorable results through significant decreases in oxidative stress biomarkers such as lactate dehydrogenase (LDH) after measuring them in blood samples at different post-PCI times [9]. It should be mentioned that molecules derived from lipid peroxidation are one of the main used biomarkers. Following Vit C administration, both a significant decrease in hydroperoxides concentration at 48 h post reperfusion [16] and a decrease in 8-isoprostanes after 6–8 h post-PCI were shown [64]. Another three trials showed a significant decrease in the levels of 8-hydroxy-2-deoxyguanosine after 1 h [65,66], 2 h [66], or 6 h [67] post-PCI. Basili et al. also showed a significant decrease in 8-iso-prostaglandin F2alpha at 6–8 h after PCI [65]. However, Guan et al. analyzed biomarkers in urine samples, revealing that there were no significant differences between the group of patients that received Vit C and the placebo group when measuring 8-hydroxy-2-deoxyguanosine for different periods of time in the first 5 h after PCI [68].

In addition, an increase in the antioxidant response has also been observed after Vit C administration during the first hours after PCI, with elevated serum ascorbate levels [64,69], a higher ferric reducing ability of plasma (FRAP) [64,69], and an increased total antioxidant capacity at 48 h after reperfusion, but not 1 month later [16]. However, the GSH/GSSG ratio decreased significantly in the groups supplemented with Vit C, having GSH levels half of those of the placebo group at 6–8 h after reperfusion [64,69].

Another parameter that can be affected by Vit C is related to inflammation, as Khan et al. [9] highlighted in their systematic review, wherein the authors suggest that the inflammatory biomarkers that have been measured in three clinical trials with Vit C after PCI, together with their results, give rise to lower levels of Thromboxane B2 (TxB2), soluble NOX2-derived peptide *(sNOX2*-*dp)*, soluble CD40 ligand *(sCD40L),* and platelet CD40L, but not high-sensitivity C-reactive protein (hs-CRP) and tumor necrosis factor alpha (TNFα) when measured within a short time frame after PCI [9].

Finally, we can conclude that the use of Vit C works to reduce the levels of oxidative stress after PCI, either by decreasing the levels of free radicals, or by enhancing the antioxidant system. However, this does not necessarily mean the obtaining of better outcomes in patients or a smaller infarct size. Other biomarkers should be analyzed together to obtain more certain evidence on the status of the patients’ heart to give some clues of the clinical outcome.

#### 3.1.2. Beneficial Effects in Cardiac Function Parameters

After seeing the results of different studies wherein Vit C has been included as a treatment for AMI, one of the most outstanding aspects is represented by the beneficial effects on the parameters of cardiac function, especially in relation to the LV, such as LVEF, LV fractional shortening (LVFS), LV isovolumic relaxation time (IVRT), cardiac output, stroke volume, and ratio of early to late ventricular filling (E/A ratio). When cardiomyopathy is induced with Doxorubicin, for example, there is a loss of cardiac function and structure. These effects include an increased heart rate, alteration in the structure of cardiomyocytes, the formation and disorder of vacuoles, loss of myofibrils, alteration in the shape of the nucleus, changes in systolic function (decreases LVFS and LVEF), and compromised relaxation and filling of the LV (increases the IVRT and E/A ratio) [9,70]. A quite recent in-vivo study showed that Vit C protects against these changes and increases survival. Indeed, the heart rate decreased, cardiac function was improved by increasing LVFS and LVEF, and protective effects were evidenced, since the formation of vacuoles was decreased and the myofibrils remained unchanged. In addition, the nucleus returned to its normal morphology and fibrosis decreased [70]. Moreover, another study suggested that Vit C has a possible functional effect because it improves the change in LVEF, and that this therapy effectively ameliorates the persistent LV impairment [64,69]. However, despite the good results, these are still not homogeneous and the exact mechanisms of action of Vit C on functional parameters have yet to be elucidated [64].

#### 3.1.3. Myocardial Damage Amelioration

A number of biomarkers in the circulation can be used to evidence myocardial injury and cell death, such as cardiac troponin I (cTnI), cardiac troponin T (cTnT), and creatine phosphokinase MB isoenzyme (CK-MB). Changes of phosphorylation of cTnI and cTnT alter sarcomeric function [71], and both are the best options to measure myocardial injury while CK-MB is less sensitive and less specific; however, it is still useful [72]. After restoring blood flow with PCI, several studies have shown an elevation of troponins and increased CK-MB. Treatment with Vit C reported positive outcomes for reducing troponin and CK-MB levels, although the results are conflicting as others studies failed to show a benefit [9,73].

#### 3.1.4. Infarct Size

Reducing infarct size after an episode of I/R is a widely studied target in cardiac pathophysiology. Although progress has been made in therapies, there are multiple pathways that favor an increase in the infarct size, including some that may not have been discovered yet. Cardiomyocyte cell death through apoptosis, necrosis, pyroptosis, autophagy, or ferroptosis is a central target to reduce infarct size [14,39]. However, there is still much to be elucidated, and the results are not of total consensus. One of the therapies used to reduce the infarct size is Vit C due to its capacity to act as a ROS scavenger and reduce oxidative stress. Moreover, it can confer cardioprotection [73]. However, another study has shown that Vit C, given prior to reperfusion at enough dosing to reach plasma levels over 10 mmol/L, did not show a significant difference in infarct size [69]. This discrepancy may be due to the fact that there are not many studies of Vit C in humans (even though it has been shown to be harmless at high concentrations) (Table 1) and the lack of consideration of basic aspects such as the mechanistic approximation of the drug and its pharmacokinetics properties [14]. Thus, high Vit C doses (e.g., 10 mmol/L plasma levels, see below) are capable of reducing O_2_·^−^ production, though is not feasible in a setting of I/R to achieve the scavenging of ·OH derived from the activation of Fenton reaction occurring in this clinical model.

### 3.2. Antioxidant Mechanism of Vitamin C

The best-known antioxidant mechanism of Vit C corresponds to that of ROS scavenger. However, Vit C must reach a plasma concentration of 10 mmol/L to displace the reaction of the superoxide anion radical with nitric oxide, which acts at a rate 10^5^ times greater than the reaction between ascorbic acid and superoxide anion radical [74,75]. Regarding the other effects of Vit C, in addition to scavenge ROS, it exerts a modulatory activity on enzymes, thus indirectly decreasing the formation of ROS (Figure 3). Within these modulations, Vit C exerts a down-regulation on the activity of enzyme NOX [76], which is present in the endothelium, leukocytes, and myocardium, being responsible for producing O_2_**^.^**^_^. Vitamin C can also inhibit the activation of the NF-κB pathway [77], thus modulating the formation of cytokines that amplify the inflammatory response that normally promotes the arrival of more leukocytes and thus increases ROS formation and damage when this pathway is active. Vitamin C also prevents the uncoupling of the eNOS enzyme by stabilizing tetrahydrobiopterin (BH4) along with preventing its oxidation [78] (Figure 3). Normally, when BH4 is oxidized, it causes eNOS to uncouple and begin to synthesize radical O_2_**^.^**^_^ instead of NO**^.^**, thus enhancing oxidative stress [79]. Moreover, impaired microcirculatory reperfusion is improved by Vit C [65].

In addition, Vit C enhances the antioxidant response where it allows for the recycling of alpha-tocopherol in the interphase of lipid-aqueous compartments [80], lipid bilayer [81], and erythrocytes [82]. It has been described as one of the most powerful antioxidants, also being its most active form within the group of molecules of Vit E [83] (Figure 3). In addition, it has been reported that the use of oral Vit C is able to restore the SOD enzyme to normal levels in patients who have suffered AMI and have been thrombolysed to reperfuse the heart [63]. However, the mechanisms remain to be elucidated.

### 3.3. Considerations on the Use of Vitamin C

Despite the previously mentioned mechanisms, the use of Vit C has not produced the expected results in patients undergoing reperfusion by PCI, which can be analyzed from various edges, being necessary to take into account a mechanistic survey of Vit C and the complexity of myocardial reperfusion damage.

To start, it should be mentioned that a plasma Vit C concentration of 10 mmol/L is needed so that it can react with the superoxide anion radical, a concentration solely achieved by intravenously administration, since pharmacokinetic studies have shown a plasma concentration of only approximately 0.08 mmol/L at steady state when Vit C is orally administered in dose ranges between 200 and 2500 mg/day [52,84]. In addition, high doses of Vit C appear to have a positive safety profile, though it should be avoided in patients with renal function impairment or glucose-6-phosphate dehydrogenase deficiency [52,85]. It is also important to consider the time in which Vit C is administered, since immediately after starting the reperfusion is when the ROS burst occurs, so a later administration could not meet the objective of ROS scavenging and thus the outcomes proposed for the patients [59].

It is of interest that Vit C can also act as a pro-oxidant species, especially in the presence of metal ions. At high concentrations, Vit C is capable of reducing Fe^3+^ to Fe^2+^, the latter being an extremely dangerous ion, as it is found either in a free form or not associated with proteins such as ferritin in the cell, and promotes the formation of large amounts of **^.^**OH via Fenton reaction [86], thus increasing oxidative stress and decreasing the concentrations of Vit C [52]. This is important in our setting of study, since, during heart I/R, there is a mobilization of iron that causes an increase in labile iron within the cells that could therefore interact with ascorbic acid and enhance the damage.

In addition, it is important to mention that Vit C, after being oxidized, is reduced again through the oxidation of GSH, a process which can occur directly or mediated by enzymes (Figure 3). This has been described in both human erythrocytes [87] and bovine aortic endothelial cells [88]. Therefore, it has been previously hypothesized that the use of high doses of Vit C could trigger GSH depletion due to the high recycling rate that is being exerted due to the constant oxidation of Vit C during reperfusion, either for acting on the ROS burst, or for the free iron mobilized during the I/R. This is supported by previous reports of a significant decrease in the GSH/GSSG ratio in human erythrocytes in clinical trials where high doses of Vit C were used after PCI [64,69].

Finally, a couple of years ago, some authors hypothesized that a multitarget therapy is necessary to be able to fully achieve a benefit in patients subjected to cardiac reperfusion, since AMI is a multifactorial process, wherein cardiomyocytes die through various pathways, such as apoptosis, necrosis, autophagy, and necroptosis [11], and where, with the passing of time, the list is enlarged, describing new pathways that could be triggering cell death after the reperfusion injury, such as ferroptosis [39,89] and pyroptosis [90]. Furthermore, not only cardiomyocytes are affected, but also other heart cell types, such as fibroblasts, immune cells, endothelial cells, and platelets [11], and targeting only one mechanism at a time may be insufficient to produce a strong and robust effect in clinical situations where many uncontrolled variables usually coexist. Therefore, the use of Vit C as a monotherapy would not be enough to reduce myocardial reperfusion damage, given the multifactorial process of damage. Vitamin C would only reduce oxidative stress under certain conditions and would not act on all the factors involved, such as the increased iron concentration. The entire cascade of damage results from a great increase in oxidative stress that can finally induce ferroptosis, as well as GSH depletion, probably caused by Vit C itself, all of which can weaken the antioxidant response. Due to this, therapies that are directed on different targets that establish an additive or synergistic effect could be the best option to finally reduce the MIRI and the infarct size [11].

**Table 1:** Results of the use of Vit C alone or in association with other antioxidants.

Model	Dose	Results	Ref.
**Human Models**			
Human (in vivo)	16.6 mg/min, over 1 h before PCI	Better preservation of CF No changes in HR, cTnT and MAP Better perfusion ↓ CK-MB levels ↓ Oxidative stress	[65]
Human (in vivo)	500 mg, twice a day for 5 days before analysis	↑ SOD activity ↑ Thiol levels ↓ XO activity ↓ MDA	[63]
Human (in vivo)	Multivitamin therapy Before reperfusion: Vit C, 1 g in IV bolus. After reperfusion (daily, for 1 month, via oral): Vit C 1 g, Vit A 50,000 Unit, Vit E 300 mg	Better preservation of cardiac function ↓ Oxidative stress ↑ Antioxidant status	[16]
Human (in vivo)	Initial dose of 2000 mg followed by a constant infusion at 20 mg/min before PCI	No suppression of oxidative stress	[68]
Human (in vivo)	1 g/L at 24 mg/min IV infusion	Better preservation of CF cTnT was similar between control and Vit C group ↓ Oxidative stress	[66]
Human (in vivo)	IV infusion of 320 mM at a flow rate of 10 mL/min during the initial hour and at 3 mL/min during the following 2 h. After the primary PCI, oral doses of Vit C (500 mg/12 h) and α-tocopherol (400 IU/day) for 84 continuous days	No significant difference in infarct size between the groups Better preservation of CF No changes in CK-MB ↑ FRAP levels	[69]
Human (in vivo)	Initial dose of 3 g IV before PCI and 100 mg of intracoronary Vit C during PCI	↓cTnT and CK-MB levels	[73]
Human (in vivo)	Initial unique oral dose of *α*-tocopherol (800 IU) and IV infusion of Vit C (320 mM) infused at a 10 mL/min flow rate during the first hour and at a 3 mL/min rate during the following 2 h. After the PCI, oral doses of Vit E (400 IU/day) and Vit C (500 mg/12 h) were taken by the patients for 84 continuous days.	Better preservation of CF No differences in CK-MB ↑ FRAP levels	[64]
Human (in vivo)	3 g IV within 6 h before PCI	↓ cTnT and CK-MB levels ↓ Oxidative stress	[67]
**Animal models**			
Adult mongrel dogs (in vivo)	100 mg/kg of Vit C was administered just before reperfusion	↓ Mortality in group of supplemented dogs ↑ GSH/GSSG ratio No significant changes in activities of GPX and GR	[91]
Domestic pigs (in vivo)	Combined treatment of 4.4 g of Vit C (about 0.1 gm/kg) and 12 g of Vit E acetate was infused	↓ Infarct size, but just reached the border of significance	[92]
Langendorff model using isolated rat hearts (ex vivo)	At the time of reperfusion one group was infused with 1 mM of AA and another group with 1 mM of AA plus 1 mM of GSHme	**AA** Slightly ↓ myocardial TBAR contents	[93]
		**AA plus GSHme** ↑ GSH content HR and CF were recovered ↓ Incidence of VF ↓ Myocardial CK loss ↓ Myocardial TBARS content ↓ Myocardial nitrotyrosine	
Young male farm pigs (in vivo)	IV infusion of 100 mg/kg AA and 60 mg/kg DFO	The therapy did not provide significant cardioprotection in the experimental group in any of the parameters measured	[94]
Farm-raised domestic male pigs (in vivo)	One group receive AA 100 mg/kg infusion. Other group receive AA 100 mg/kg + DFO 60 mg/kg + NAC 100 mg/kg for 20 min with a 20-mg/kg maintenance dose	The therapy did not provide significant cardioprotection in the experimental group in any of the parameters measured	[95]
Langendorff model using isolated male Sprague-Dawley rats hearts (ex vivo)	Hearts were post-treated with 2 μM Vit C for 30 min after global ischemia	↓ I/R-Induced infarct area ↓ LDH activity Improved all hemodynamic variables ↑ NAD+, suggested that Vit C inhibited mPTP opening ↓ Apoptosis ↑ Oxygen consumption	[96]
**Cell Cultures**			
HeLa and MCF7 cells (in vitro)	HeLa cells incubated with 1 mM DHA for 1 h and accumulated 4 mM intracellular Vit C. Conversely, cells incubated with 1 mM Vit C for 1 h accumulated 0.2 mM intracellular Vit C *	Inhibits: TNF*α*-induced transcriptional responses mediated by NF-κB TNF-dependent nuclear translocation of NF-κB The TNF*α*-induced phosphorylation and degradation of IκBR	[77]
Neonatal rat cardiac fibroblast (in vitro)	Cells treated with Vit C in doses of 1 μM, 10 μM 100 μM, 10,000 μM	No effect in cell viability at 1 and 10 μM ↑ Cell viability but not significantly at 100 μM ↓ Cell viability at 10,000 μM	[97]
	Cells treated with different combinations of Vit C, DFO, NAC (Vit C/DFO, Vit C/NAC and Vit C/NAC/DFO), each in doses of 1 and 10 μM	↑ Cell viability only Vit C/DFO in doses of 1 μM ↑ Cell viability Vit C/DFO, Vit C/NAC and Vit C/NAC/DFO in doses of 10 μM ↓ Intracellular ROS production Vit C/NAC/DFO in doses of 10 μM	
HUVEC HCAEC (in vitro)	Cells were preloaded with AA by incubating with different concentrations of DHA for 30 min before subjecting to hypoxia	↓ Apoptosis ↓ ROS levels Prevents release of Cyt C to cytosol Stabilizes mitochondrial membrane potential Inhibits procaspase-9 and procaspase-3 activation	[98]
Neonatal rat cardiac ventricular myocytes (in vitro)	Cells were post-conditioned with normal culture medium containing 2 μM Vit C	↑ Cell viability ↓ LDH activity ↓Cytosolic Ca^2+^ overload. ↓ ROS levels Alleviated mPTP opening in cardiomyocytes Preserved ΔΨm ↑ AKT (Ser473) phosphorylation ↑ Expression of p-GSK 3*β*(Ser9)	[96]

* Probably due to conversion of Vit C to DHA under aerobic conditions: AA, ascorbic acid; CF, cardiac function; CK-MB, creatinine phosphokinase MB isoenzyme; cTnT, cardiac troponin; Cyt c, citocrome C; DHA, dehydroascorbic acid; DFO, deferoxamine; FRAP, ferric reducing ability of plasma; GPX, glutathione peroxidase; GR, glutathione reductase; GSH, reduced glutathione; GSSG, glutathione disulfide; HR, heart rate; IV, intravenously; LDH, lactate dehydrogenase; MAP, mean arterial pressure; MDA, malondialdehyde; mPTP, mitochondrial permeability transition pore; NAC, N-acetylcysteine; PCI, percutaneous coronary intervention; ROS, reactive oxygen species; SOD, superoxide dismutase; TBAR, thiobarbituric acid reactive substances; TNF, tumor necrosis factor; Vit A, vitamin A; Vit C, vitamin C; Vit E, vitamin E; XO, xanthine oxidase; ΔΨm, mitochondrial membrane potential; ↑, increase; ↓, decrease.

## 4. Cardioprotective Effects Exerted by Other Antioxidants

Over time, various antioxidants have been used to generate cardioprotection after MIRI, such as Vit E, NAC, DFO, and polyphenols, obtaining different results in in-vivo and in-vitro studies. A widely used method to study I/R is the Langendorff model (ex vivo), which basically consists of a functionally active isolated heart having a cannula inserted into its aorta so that the heart can be retrogradely perfused via the coronary circulation [99]. On the other hand, in-vivo studies have also been performed in the hearts of living whole organisms of species such as a pig, dog, rabbit, sheep, mouse, and rat [100]. Finally, cardiac tissue cell cultures have also been subjected to study the in-vitro protective effects of antioxidant in I/R settings. The main results are summarized in Table 2.

### 4.1. Vitamin E

**Figure 4:**
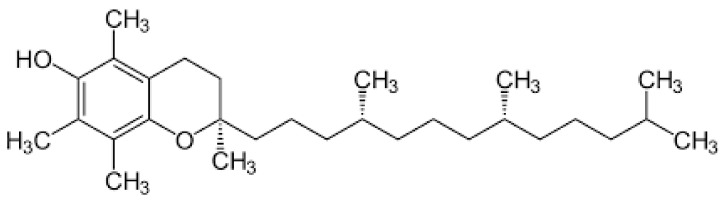
Molecular structure of α-tocopherol.

Vitamin E (Figure 4) is a group of lipid-soluble agents with antioxidant and anti-inflammatory effects, where α-tocopherol has been described as one of the most active and effective forms in terms of antioxidant power [83]. Its antioxidant mechanisms are mainly based on its ability to act as an ROS scavenger and contribute to enzymatic regulation, such as increasing the activity of glutathione peroxidase and decreasing the release of ROS through the down-regulation of NOX [76]. Regarding its anti-inflammatory mechanism, it is important to mention its property to inhibit the transcriptional activity of NF-κB factor [75,116].

Vitamin E has demonstrated cardioprotection in models of pathologies related to oxidative stress, such as arterial hypertension, AMI, and postoperative atrial fibrillation. Regarding its use to reduce damage due to myocardial reperfusion, different results have been obtained that are not conclusive, however Lassnigg et al. [94] carried out a clinical trial wherein the concentration of Vit E was normalized in patients subjected to coronary artery bypass graft surgery, valve surgery, or combined procedures, in which it had no effect on oxidative stress biomarkers or on postoperative clinical outcomes [117]. Moreover, in a recent study, the use of Vit E was able to reduce the biomarkers of oxidative stress and inflammation in a murine model of I/R along with the preservation of cardiac function [101]. This has also been used in combination with Vit C in a clinical trial [64] that reported an improvement in cardiac function and a decrease in the levels of oxidative stress in patients subjected to angioplasty.

Therefore, in summary, Vit E has showed a beneficial effect due to its radical scavenger activity when it is administered at the correct time and for an appropriate duration [118]. However, Miller et al. concluded that high doses (>400 IU/d) may increase all-cause mortality [119].

### 4.2. N-Acetylcysteine

**Figure 5:**
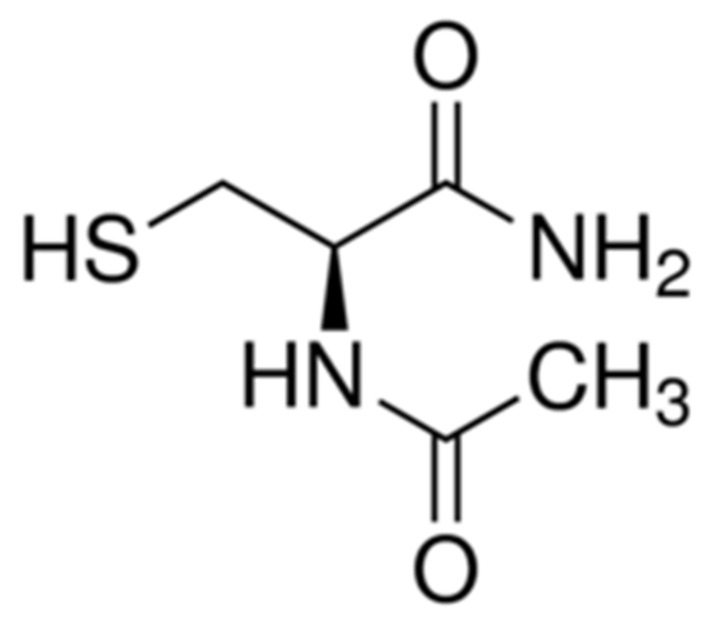
Molecular structure of N-acetylcysteine.

It is a compound having widely clinical use, having shown to be safe and well tolerated when administered orally [120]**.** Its results in cardioprotection have not been conclusive, and, at present, its mechanism of action has not been fully elucidated; however, with the passage of time, extensive evidence has been gathered that establishes Vit E as a key point, being a precursor for the synthesis of GSH and therefore an extremely important molecule for the antioxidant system. It is a compound having widely clinical use, having shown to be safe and well tolerated when administered orally [120]**.** Its results in cardioprotection have not been conclusive, and, at present, its mechanism of action has not been fully elucidated; however, with the passage of time, extensive evidence has been gathered that establishes Vit E as a key point, being a precursor for the synthesis of GSH and therefore an extremely important molecule for the antioxidant system. N-Acetylcysteine (Figure 5) is a compound having widely clinical use, having shown to be safe and well tolerated when administered orally [120]**.** Its results in cardioprotection have not been conclusive, and, at present, its mechanism of action has not been fully elucidated; however, with the passage of time, extensive evidence has been gathered that establishes Vit E as a key point, being a precursor for the synthesis of GSH and therefore an extremely important molecule for the antioxidant system. Moreover, NAC have indirect action as a metal ion chelator [121] and an ROS scavenging effect, along with the ability to inhibit NF-κB [122].

In clinical trials, NAC has been administered to patients undergoing angioplasty, where the results in reducing MIRI have been inconclusive. In an LIPSIA-N-ACC trial, NAC infusion was used during and after reperfusion, showing insignificant effects on the size of the infarct, but a decrease of 20% in oxidative stress parameters, measured from oxidized proteins and lipoproteins [103]. In another study, where patients received NAC infusion with nitroglycerin and streptokinase, a better preservation of the left ventricular function and a decrease in oxidative stress biomarkers were obtained [104]. Nozari et al. performed a randomized, double-blind, placebo-controlled trial, wherein they administered NAC before and during reperfusion. This resulted in a significant decrease in hs-TNT levels in patients supplemented with NAC versus the placebo, along with a higher percentage of patients with TIMI 3 flow in those who received the drug [105]. However, this study did not measure infarct size or other oxidative stress biomarkers. The NACIAM study was a randomized, double-blind, placebo-controlled, multicenter trial that administered a total of 29 g of intravenous NAC infusion during the first 48 h after angioplasty in combination with nitroglycerin, showing a decrease in the infarct size, but no difference in functional parameters such as LVEF and end-systolic volumes [123]. Moreover, NAC was found to confer cardioprotection in other clinical models of I/R, such as in the study of Ozaydin et al., which was conducted as a prospective, randomized, placebo-controlled pilot study to prevent postoperative atrial fibrillation by administering NAC infusion, reporting a decreased incidence of postoperative atrial fibrillation in which adverse effects associated with NAC were found [106].

The side effects of NAC remains unclear, but so far NAC has shown no relevant adverse effects and is cheap and easily available [124,125].

### 4.3. Deferoxamine

**Figure 6:**
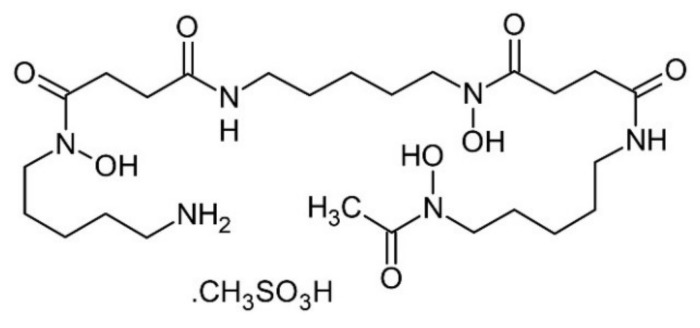
Molecular structure of deferoxamine mesylate.

Iron plays a fundamental role in MIRI, as an increased plasma concentration of this metal ion has been demonstrated after heart reperfusion. Ferrous iron has been described as being part of the labile iron pool which is capable of reacting with hydrogen peroxide and producing the dangerous ·OH, thereby triggering extensive oxidative damage. Furthermore, in recent years, evidence has shown its importance in a new, non-apoptotic cell death pathway called ferroptosis, which has a highly inflammatory component and can spread to neighboring cells [126]. It has been described in animal models of I/R [89], in which Tang et al. demonstrated that ferroptosis occurs during reperfusion and not in ischemia in a rat heart model of I/R [127]. Therefore, the use of a chelating agent for this metal ion is relevant when it is intended to develop a therapy designed to lower MIRI. Some clinical trials have been carried out using DFO (Figure 6) during myocardial reperfusion. For example, Paraskevaidis et al. administered 4 g of DFO dissolved in 250 mL of 5% dextrose solution continuously for 8 h in patients undergoing coronary artery bypass grafting, obtaining in the group treated with DFO a complete suppression of the formation of ROS after surgery and a significant improvement in the LVEF, compared to the placebo group. The increase in the LVEF was observed in a much more representative way in the group of patients with a worse LVEF [107]. Subsequently, Chan et al. administered 500 mg of DFO 5 to 10 min before PCI, followed by 50 mg/kg over 12 h infusion which achieved a significant decrease in serum iron and plasma F_2_-isoprostane levels when compared to the placebo during the first hours. However, the infarct size did not decrease significantly [108].

In addition, DFO is commonly used as a treatment for iron poisoning and other pathologies as several studies have determined that acute DFO administration is safe. However, prolonged intravenous doses might cause cardiovascular, pulmonary, and auditory toxicity, as well as an increase in the risk of infection [128,129].

### 4.4. Polyphenols and Other Antioxidant Compounds

**Figure 7:**
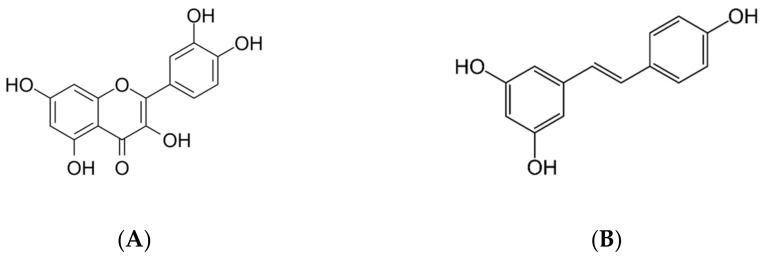
Molecular structures of two polyphenols: quercetin (**A**) and resveratrol (**B**).

Polyphenols are compounds present in a diet, especially in fruits, vegetables, and red wine. They are classified as flavonoid, such as flavonols or anthocyanins and non-flavonoid such as stilbenes [130]. Their structure is characterized by an aromatic ring and contains one or more hydroxyl substituents and can be a simple molecule or highly polymerized compounds. Polyphenols have shown promising effects in preventing various diseases due to their ability to act as scavengers of reactive nitrogen and oxygen species and their role in improving inflammation and activating antioxidant enzymes and metal chelators [131]. Recently, research on polyphenols has been increasing due to their pharmacological characteristics. Most studies have focused on their various bioactivities, such as their antioxidant, antitumor activity, anti-inflammatory effects, and free radical scavenging [132,133,134]. (Figure 7). Polyphenols have shown promising effects in preventing various diseases due to their ability to act as scavengers of reactive nitrogen and oxygen species and their role in improving inflammation and activating antioxidant enzymes and metal chelators [131]. Recently, research on polyphenols has been increasing due to their pharmacological characteristics. Most studies have focused on their various bioactivities, such as their antioxidant, antitumor activity, anti-inflammatory effects, and free radical scavenging [132,133,134].

There are other important properties that can be relevant, especially in the heart, to confer cardioprotection, but this effect cannot be attributed to all polyphenols, since some of them had no significant effect on the resultant damage and others displayed undesired side effects [135,136,137]. Nevertheless, many natural types produce positive effects on the cardiovascular system and are characterized by their ability to scavenge oxygen-free radicals, maintain NO concentration, inhibit an excessive immune innate response, improve endothelial function, and reverse hyperlipidemia [138].

It is known that the inflammatory response, which includes neutrophil infiltration, plays an important role in the I/R process. NLRP3 (NALP3) inflammasome, which contains pro-Caspase1, produces an inflammatory response by the production of IL-1. Inhibition of IL-1, as well as IL-18, reduces MIRI [114,139]. In previous studies, resveratrol, a stilbene-derived flavonol, has shown cardioprotective effects and beneficial effects against MIRI [140,141], and Dong et al. confirmed its role in inhibiting the innate immune pathway [114].

In addition, the association between polyphenols and Vit C produces an antioxidant synergy, since Vit C protects the flavonoids from oxidative degradation and flavonoids, in turn, act as antioxidants and iron chelators. Consistent with this view, the metal ion–flavonoid complexes retain the antioxidant activities of the flavonoids and increase the free radical scavenging activities [136,142].

With regard to the use of polyphenols, care must be taken with the doses administered and the possible toxic and deleterious effects that they can cause, as in the case of C3OG, which, at the highest concentrations (50–100 µmol/L), does not ensure a greater effect but rather induces toxicity. Another difficulty is translating laboratory studies of polyphenols into clinical practice, given the existence of a certain inconsistency between preclinical and clinical trials, most likely dictated by the fact that metabolism and physiological concentrations were not taken into account [130,138].

Moreover, despite resveratrol being one of the most studied polyphenols, there are few studies about its toxicity, and the follow-up time is short. These studies have shown that high doses have a toxic effect due to its hormetic property because resveratrol acts as a pro-oxidant molecule [143,144]. The effects depend on the time course of administration, dosing, study design, interactions with other drugs, and on the characteristics of the enrolled patients [144].

## 5. Towards a Potential Synergistic Cardioprotection Achieved by Combined Antioxidants

Myocardial ischemia reperfusion injury can produce extensive damage because it acts in multiple ways, acutely and chronically. Although, in recent years, there have been several studies on ischemic conditioning (pre- and post-conditioning), they have been disappointing in their clinical application [145]. Because of this, other ways to achieve cardioprotection have been studied, and a cost-effective study has established the positive economic consequences of the use of a cardioprotective therapy in patients with AMI involving endogenous cardioprotection strategies, beta-blocker therapy, or even mitochondria-targeted cardioprotection strategies [11,145,146], which makes it a very striking strategy not only for its effects at the tissue level. According to Davidson et al., a multitarget cardioprotective therapy is defined as the additive or synergistic cardioprotective effects of multiple agents or interventions directed to distinct targets [11] and that, in the future, this option should be considered as a parallel treatment to the restoration of blood flow after ischemia. A combination of drugs such as Vit C and Vit E has been carried out in MIRI but without the expected success, as had occurred with this association in causing a significant decrease in blood pressure [147,148]. Other multitarget studies have been successful [145], suggesting that a combination of antioxidants and other antihypertensive drugs are useful for cardiovascular diseases [148]. In light of this, we focus on the combination of Vit C acting as an ROS scavenger, DFO acting on iron metabolism (reducing ·OH formation), and NAC acting as a GSH precursor (Figure 1) as a promising combined antioxidant therapy that should be evaluated in randomized clinical trials [148].

## 6. Discussion

Oxidative stress has been one of the most studied mechanisms within the pathophysiology of MIRI, and this has led to antioxidant-based therapies. Thus, to abrogate the consequences of this injury, Vit C and other antioxidants have been proposed as alternatives. However, the achievements in human models have not been consistent with those of experimental studies. In the case of Vit C, firstly, it could be argued that there are limitations in using a dose known to lead to superoxide scavenge, as high plasma Vit C concentrations generate an iron-dependent pro-oxidant effect, whereas a weak effect can be expected from low doses. In addition, there is a difficulty of comparison between studies, given the existence of a wide variability of designs present in the state of the art, which differ in the model, doses, forms of administration, and the time course of Vit C administration (Table 1). In addition to the before-mentioned studies, there are a few which relate the effects of therapy with the plasma levels of the drug reached in patients, which has led to obscuring the analysis of the concentration: effect ratio. Experimental data induces us to think of a biphasic action of Vit C, achieving a beneficial effect until intermediate concentrations, but this effect has been lost at 10 mmol/L and replaced by a pro-oxidant one.

Of note is the strength given by the beneficial functional actions that have already been previously reported in patients, both from Vit C and other antioxidants. In addition, it is also important to take into consideration that the nature of the clinical model being studied requires a brief and unique exposure to block the damage caused by ROS during the first minutes of reperfusion, thus avoiding the risk of adverse events. Numerous studies have contributed to demonstrate the safety of Vit C when used in wide ranges of doses, either alone or combined with other antioxidant agents. As the diversity of antioxidants offer various kinds of antioxidant mechanisms, a joint cardioprotective effect derived from the addition of the individual biological actions known to occur in the monotherapies should be expected. Finally, even though MIRI has been studied for decades, which is a highly prevalent process, given the epidemiology of acute myocardial infarction worldwide, it has not yet been possible to establish a treatment that can fully demonstrate cardioprotective efficacy, and the present study provides an underexplored opportunity for progress in this line of research.

## 7. Concluding Remarks and Future Perspectives

It is well recognized that Vit C is involved in the antioxidant effect against oxidative stress related diseases as those derived from I/R events, such as AMI followed by PCI. Although considerable effort has been previously devoted to prevent the reperfusion damage by antioxidants, the results remain disappointing. This could be due to lack of accurate clinical trials, the need for a better knowledge of pharmacodynamic and pharmacokinetic properties of Vit C and other antioxidants, or the required improvement of flawed study designs. Partly successful individual effects of antioxidant monotherapies have been reported in clinical studies. Nevertheless, the complexity of a pathology such as AMI followed by PCI demands a specific design of appropriate strategies aimed to abrogate the pathophysiological injurious cascade causing cell death with the aid of an association of antioxidants. Thus, we hereby propose a novel multitarget therapy based on the antioxidant and pleiotropic properties of Vit C, Vit E, or even polyphenols, and NAC, accounting for the scavenging of reactive oxygen species and the generation of reduced glutathione. Particularly relevant is the view that iron chelation (DFO) can lead to a reinforcement of cardioprotection in mitigating the harmful OH generation and the occurrence of ferroptosis-induced cell death, recently recognized as a new form of cell death. The aim of this association in clinical therapeutic perspectives for humans is to achieve a synergistic protection against I/R injury, thereby diminishing the final infarct size to improve the clinical outcome of AMI patients subjected to PCI through the administration of safe, low-cost, and easily available pharmacological agents.

## Figures and Tables

**Table 2:** Results of the use of different antioxidants to reduce MIRI.

Antioxidant		Model	Dose	Results	Ref.
Vit E α-tocopherol (C_29_H_50_O_2_)		C57BL/6 mice (in vivo)	2.5 mg/kg BW in 0.8% DMSO 2 h prior to surgery, immediately after PCI, and twice per day for three consecutive days	↓ Infarct size ↓ ROS and lipid oxidation ↓ MPO activity ↓ Neutrophil infiltration Prevented pathological changes	[101]
		Langendorff model using male Hartley Guinea pigs hearts (ex vivo)	100 μM	QT segment recovered 10%	[102]
NAC (C_5_H_9_NO_3_S) NAC (C_5_H_9_NO_3_S)		Human (in vivo)	IV bolus of 1200 mg before PCI and 1200 mg IV twice daily for the 48 h after PCI (total dose 6000 mg)	↓ Oxidative stress It does not provide an additional clinical benefit to placebo with respect to patients undergoing PCI. No adverse effects.	[103]
		Human (in vivo)	Patients with AMI received 15 g infused over 24 h + IV NTG and streptokinase	↓ Oxidative stress. NAC and GSH concentration were correlated. ↓ MDA concentrations over the first 8 h of treatment. Better preservation of LV function. No adverse effects.	[104]
		Human (in vivo)	NAC 100 mg/kg bolus followed by intracoronary NAC 480 mg during PCI then IV NAC 10 mg/kg for 12 h	↓ Peak hs-TnT level after PCI. Difference in peak CK-MB was not statistically significant. No adverse effects	[105]
		Human (in vivo)	Infusion of 50 mg/kg, followed by IV infusion for 48 h after the operation at a dose of 50 mg/kg/day	↓ Rate of atrial fibrillation in the NAC group.	[106]
DFO (C_25_H_48_N_6_O_8_)		Human (in vivo)	4 g were infused for 8 h	Prevented ROS production Improved LVEF No major cardiac event was reported with long term administration	[107]
		Human (in vivo)	500 mg 5 to 10 min before PCI, followed by 50 mg/kg over 12 h	↓ Serum iron and plasma F2-isoprostane levels during the first hours. No changes in the infarct size.	[108]
Polyphenols (Flavonols)	Quercetin (C_15_H_10_O_7_)	Cells suspension of rats (Wistar strain) Thymocytes (in vitro)	2 mL cell suspension in a 10 mL test tube	Protective effect on the cells suffering oxidative stress and cells suffering from intracellular Ca^2+^ overload. ↓ Cell death	[109]
		Langendorff model using male Wistar rat hearts (ex vivo)	15 µM	Improvement in the functional parameters of the heart (LVDP and contractility) ↓ End-diastolic pressure.	[110]
		Human (in vivo)	500 mg twice daily for 1 month	↓ Inflammation	[111]
Polyphenols (Stilbenes)	Resveratrol (C_14_H_12_O_3_)	Male rats (Sprague-Dawley) (in vivo)	100 µM	↓ Infarct size, ↓ Myocardial apoptosis ↓ NF-κB expression ↓ Neutrophil infiltration ↓ TNF-α levels ↓ Cardiac dysfunction ↓ Activity of serum CK-MB and LDH level ↓ MDA levels ↑ Antioxidant enzymes activities ↑ Nrf2 and HO-1	[112,113]
		Male rats (Sprague-Dawley) (in vivo)	2.5, 5, and 10 mg/kg	↓ Necrotic area ↓ TnT and CK-MB release ↓ IL-1β and IL-18 release ↓ Myocardial NALP3 expression Inhibits I/R-mediated myocardial Caspase1 expression.	[114]
Polyphenols (Anthocyanins)	C3OG (C_21_H_21_O_11_^+^, Cl^−^)	Langendorff model (ex vivo) using hearts from male rats (Wistar)	20 μM	↓ Cardiomyocyte death ↓ LDH levels Protection against apoptosis induced by I/R Cytochrome c-reducing activity Stimulation of mitochondrial respiration after ischemia	[115]
			40 μM	No significant changes compared to 20 μM of C3OG	
	P3OG (C_21_H_21_O_10_^+^)	Langendorff model (ex vivo) using hearts from male rats (Wistar)	20 μM	Cardiomyocyte death was not statistically different from the I/R control group ↑ LDH activity than in the control group and similar to the I/R group	
			40 μM	No significant changes compared to 20 μM of P3GO	

AMI, acute myocardial infarction; C3OG, Cyanidin-3-O-glucoside; CK-MB, creatinine phosphokinase MB isoenzyme; DFO, deferoxamine; DMSO, dimethylsulfoxide; GSH, reduced glutathione; HO-1, heme oxygenase-1; IL, Interleukin; IV, instravenously LDH, lactate dehydrogenase; LV, left ventricule; LVDP, left ventricular diastolic pressure; LVEF, left ventricular ejection fraction; MDA, Malondialdehyde; MPO, Myeloperoxidase; NAC, N-acetylcysteine; NALP3, NLRP3 inflammasome; NF-κB, nuclear factor kappa-light-chain-enhancer of activated B cells; NTG, nitroglycerin; Nrf2, nuclear factor-erythroid 2-related factor 2; P3OG, Pelargonidin-3-O-glucoside; PCI, percutaneous coronary intervention; ROS, reactive oxygen species; TNF-α, tumor necrosis factor alpha; TnT, troponin T; Vit E, vitamin E; ↑, increase; ↓, decrease.

## Data Availability

Not applicable.

## Abbreviations

AMI	Acute myocardial infarction
AREs	Antioxidant response elements
ATP	Adenosine triphosphate
BH4	Tetrahydrobiopterin
C3OG	cyanidin-3-O-glucoside
CAT	catalase
cTnI	troponin I
cTnT	cardiac troponin T
CK-MB	creatinine phosphokinase MB isoenzyme
DFO	deferoxamine
DHA	dehydroascorbic acid
FRAP	ferric reducing ability of plasma
Gclc	Glutamate-Cysteine Ligase Catalytic Subunit
Gclm	Glutamate-Cysteine Ligase Modifier Subunit
GPXs	glutathione peroxidases
GR	glutathione reductase
GRXs	glutaredoxins
GSH	reduced glutathione
*hs*-*CRP*	high-sensitivity C-reactive protein
HO-1	heme oxygenase-1
H_2_O_2_	hydrogen peroxide
I/R	ischemia/reperfusion
IVRT	LV isovolumic relaxation time
Keap1	Kelch-like ECH-associated protein 1
LDH	lactate dehydrogenase
LV	left ventricle
LVEF	LV ejection fraction
LVFS	LV fractional shortening
MDA	malondialdehyde
MIRI	myocardial ischemia reperfusion injury
MPO	myeloperoxidase
mPTP	mitochondrial permeability transition pore
NAC	N-acetylcysteine
NADPH	nicotinamide adenine dinucleotide phosphate
NALP3	NLRP3 inflammasome
NF-κB	nuclear factor kappa-light-chain-enhancer of activated B cells
NO^.^	nitric oxide radical
NOO^.^	nitrogen dioxide radical
NOX	NADPH oxidase
Nrf2	nuclear factor-erythroid 2-related factor 2
^1^O_2_	oxygen singlet
O_2_**^.^**^_^	superoxide anion radical
OH	hydrogen peroxide
ONOO-	peroxynitrite anion
P3OG	pelargonidin-3-O-glucoside
PCI	percutaneous coronary intervention
PRXs	peroxiredoxins
ROS	reactive oxygen species
SODs	superoxide dismutases
*sCD40L*	soluble CD40 ligand
*sNOX2*-*dp*	soluble NOX2-–derived peptide
SVCT1	sodium-ascorbate co-transporters 1
SVCT2	sodium-ascorbate co-transporters 2
*TNFα*	tumor necrosis factor alpha
TRXs	thioredoxins
TxB2	Thromboxane B2
un-eNOS	uncoupled endothelial nitric oxide synthase
Vit C	vitamin C
Vit E	vitamin E
XO	xanthine oxidase
